# Sleep quality and its clinical associations in trichotillomania and skin picking disorder

**DOI:** 10.1016/j.comppsych.2020.152221

**Published:** 2021-02

**Authors:** Elizabeth Cavic, Stephanie Valle, Samuel R. Chamberlain, Jon E. Grant

**Affiliations:** a University of Chicago, USA; b Cambridge University, United Kingdom

**Keywords:** Trichotillomania, Skin-picking disorder, Sleep quality, Stress

## Abstract

**Background:**

Trichotillomania (TTM) is characterized by recurrent hair pulling and associated hair loss. Skin picking disorder (SPD) is characterized by recurrent skin picking and associated scarring or tissue damage. Both disorders are also accompanied by psychological distress and poor sleep. Very little, however, is known about lifestyle variables that may contribute to symptom severity in these disorders.

**Methods:**

We recruited 87 adults as part of a cross-sectional study of 3 groups (TTM, SPD, and non-affected). Clinical subjects (*n*=69) were compared with controls (*n*=18) on sleep quality as measured by Pittsburgh Sleep Quality Index (PSQI). We used partial least squares regression to identify which variables were significantly associated with poor sleep quality among those participants with TTM or SPD.

**Results:**

Clinical subjects had significantly poorer sleep quality than controls. Sleep quality was significantly related to older age, worse perceived stress, lower distress tolerance and greater impulsivity in adults with BFRBs. Poor sleep quality was associated with worse hair pulling symptom severity but not skin picking severity. Higher levels of comorbid mental disorders was also associated with worse sleep, above and beyond the impact of these other variables.

**Conclsuions:**

Poor sleep quality appears to be related to multiple variables. Further research is needed to determine causality and to tailor treatment to specific patient needs.

## Highlights

• Trichotillomania and skin picking disorder are both associated with poor sleep quality compared to controls.
• Sleep quality is associated with age, perceived stress, distress tolerance and impulsivity.
• Poor sleep quality is associated with worse hair pulling but not worse skin picking.

## 1 Introduction

Skin-picking disorder (SPD), characterized by recurrent skin picking resulting in tissue damage, and trichotillomania (TTM), defined by a repetitive hair-pulling behavior, are both psychiatric disorders grouped under the larger conceptual umbrella of body-focused repetitive behaviors (BFRBs). In addition to the repetition of the body-focused behavior and associated damage to the affected area, these conditions often lead to clinically significant impairment or distress [1]. While these disorders are not always debilitating, they are often accompanied by increased anxiety, depression, and other psychosocial dysfunction [[1], [2], [3]].

A growing body of literature suggests that lifestyle factor may be associated with either the onset of mental health disorders or with their symptom severity [4]. In fact, the Royal Australian and New Zealand College of Psychiatrists' practice guidelines for mood disorders recommend targeting lifestyle factors (e.g., sleep, exercise, diet, smoking) before initiating pharmacotherapy or psychotherapy [5]. The role of lifestyle factors in relation to BFRBs, however, has yet be fully understood. A recent internet-based survey of one lifestyle factor (i.e. sleep) found that those with TTM or SPD reported greater sleep complaints than healthy controls even after controlling for depressive and anxiety symptoms, and that sleep problems were associated with greater symptom severity [6]. Further support for this relationship between BFRB behaviors and sleep problems can be found in the only other study we are aware of that examined sleep in SPD subjects (*n*=10) and found greater sleep difficulties and higher levels of perceived stress when compared to controls [7]. Taken together, these preliminary studies suggest that there may be a link between sleep quality, stress, and BFRB symptom severity.

In addition to the limited research about sleep and BFRBs, the topic of sleep may be particularly relevant to BFRBs for other reasons as well. Prior research has suggested that there is a direct relationship between sleep deprivation and self-reported stress and anxiety [8,9]. Second, inadequate sleep shows relationships with emotional dysregulation and impaired impulse control [10]. This link between poor sleep and impulse control may be driven in part by impaired response inhibition which has been associated with sleep deprivation [[11], [12], [13]], a potentially useful link to BFRBs given data supporting a stop signal deficit in these behaviors [14]. Overall, these studies suggest that poor quality sleep is associated with stress and impulsivity, two relevant domains in our understanding of BFRBs.

One approach to refining our treatment of BFRBs might be to better understand sleep difficulties and their clinical associations. Here, our aim was to identify clinical and demographic measures associated with sleep problems (i.e. a modifiable health behavior) in a sample of adults with BFRBs. Given the extant literature, we hypothesized that sleep problems would be associated with a greater degree of impulsivity and worse BFRB symptom severity. If data from our larger sample show sleep difficulties in people with BFRBs, and that sleep problems are associated with symptom severity, this may well lay the foundation for addressing this lifestyle factor before initiating treatment interventions for the BFRB. These findings may also add to the growing transdiagnostic research implicating sleep disturbances in mental health conditions and as such might even lead to new treatment interventions for BFRBs that are essentially transdiagnostic sleep interventions.

## 2 Methods

### 2.1 Participants

Non-treatment seeking adults, ages 18–65 with a primary and current DSM-5 diagnosis of TTM (*n*=37; 91.9% female) or SPD (*n*=32; 90.6% female) were enrolled. An additional 14 females and 4 males, ages 18–65, were recruited as non-affected controls. Participants were recruited using flyers, online advertisements, and referrals. For all participants, exclusion criteria included: unstable and/or inconsistent psychotropic medication within the past three months, or any neurological conditions that would preclude completion of neurocognitive tasks or questionnaires. Additionally, non-affected controls were excluded if they had any current or lifetime psychiatric disorder or any current or lifetime use of psychotropic medication.

Data were collected at the University of Chicago from March 2017 to September 2018. The study, and consent processes, were approved by The University of Chicago Institutional Review Board. After participants were given a comprehensive explanation of study procedures and an opportunity to ask any questions, they provided written informed consent. This research was conducted in accordance with the principles of the Declaration of Helsinki.

All participants completed a comprehensive diagnostic interview (Mini International Neuropsychiatric Interview 7.0 (MINI 7.0) [15]; BFRB diagnostic modules and symptom severity scales, which were completed by trained diagnosticians with a bachelor's degree or higher trained to reliability and supervised by a doctoral-level clinician; neurocognitive tasks from the Cambridge Neurocognitive Test Automated Battery (CANTAB; http://www.cantab.com); and self-report questionnaires regarding BFRB symptoms. The total assessment time was approximately 3–4 h completed as a single visit. Participants were compensated 75 USD for time and travel, and were permitted to take rest breaks during the procedures if needed.

### 2.2 Assessments

#### 2.2.1 Clinical features

Participants were diagnosed using DSM-5 criteria. A semi-structured interview was used to acquire demographic information and data regarding the clinical characteristics of BFRBs. The interview included questions regarding skin picking or hair-pulling behavior, including age of onset, intensity of urges, environmental or emotional triggers, and frequency and duration of the behavior.

We used the Mini-International Neuropsychiatric Interview (MINI) [15] to screen for co-occurring or lifetime psychiatric disorders. Controls were excluded at this point if they were found to be experiencing current or lifetime neuropsychiatric disorders. Family medical and psychiatric history was also assessed.

#### 2.2.2 Clinical measures

Clinical measures were used to assess symptom severity, sleep quality, as well as anxiety, stress, and impulsivity. All measures have acceptable psychometric properties (we have calculated the internal consistency measures for each of the following scale).

*Massachusetts General Hospital Hairpulling Scale (MGH-HPS).* The MGH-HPS has 7 items, and is a self-report scale that assesses severity of hair-pulling during the past week. Questions are scored from 0 (no symptoms) to 4 (severe symptoms) [16]. Cronbach's alpha = 0.971.

*Skin Picking Scale – Revised (SPS-R).* The SPS-R has 8-item, and examines several domains of skin picking and is scored from 0 (none) to 4 (extreme) [17]. Cronbach's alpha = 0.967.

*Pittsburgh Sleep Quality Index (PSQI).* The PSQI has 18-items, and is used to assess sleep disturbances and quality over the past month. The first three questions are open-ended and the remaining 15 questions deal with sleep disturbances and sleep quality [18]. A total score of greater than 5 indicates “poor” sleep quality. Cronbach's alpha = 0.711.

*Perceived Stress Scale (PSS).* The PSS has 10-item items, and assesses the degree to which respondents believe their ability to cope is outweighed by their life stressors. Total scores range from 0 to 40; a score of 0–13= low perceived stress; scores of 14–26= moderate perceived stress, and scores of 27 or greater = high perceived stress [19]. Cronbach's alpha = 0.499.

*Adult ADHD Self-Report Scale (ASRS-v1.1).* The ASRS screen for ADHD using six items [20]. For each item, the individual rates the frequency of a given difficulty or behavior on a scale of 0 (never) to 4 (very often). Each response of sometimes or greater (2 or more) on screening items 1–3 equates to one point; each response of often or greater (3 or more) on screening items 4–6 results in a point. A total score of four or greater indicates probable ADHD. Cronbach's alpha = 0.867.

*The Distress Tolerance Scale (DTS).* The DTS consists of 15 items and assesses emotional distress and ability to tolerate distress [21]. Participants are asked as to how much they agree with each statement from 1 (strongly agree) to 5 (strongly disagree). Cronbach's alpha = 0.909.

*Barratt Impulsiveness Scale 11 (BIS-11).* The BIS-11 is a 30 question self-report questionnaire examining impulsivity [22] The BIS-11 uses a 4-point scale for each response (responses are: rarely/never, occasionally, often, or almost always/always). The BIS-11 responses are used to generate three subscores: motor, non-planning, and attentional impulsivity. Cronbach's alpha = 0.682.

*Stop Signal Task (SST).* We had each participant undergo cognitive testing with the SST to assess objectively their inability to inhibit a prepotent motor response. In this task, the participant is taught to respond to a stimulus, and after practicing, the participant is instructed that they should withhold a response if there is an auditory signal after the stimulus is shown. The primary measure of interest on the task is the stop signal reaction time (SSRT), which assesses inhibitory control [23]. Longer SSRTs equate to worse inhibitory control. The version of the SST deployed was the conventional CANTAB version. SSRT was estimated by subtracting average Stop Signal Delay (SSD) from median no-signal Reaction Time (RT); and the program was titrated to performance (50% probability of successful inhibition) using staircases.

### 2.3 Data analysis

All groups and subgroups were compared using two-sample, two-tailed z-tests to ensure demographic consistency across groups. TTM and SPD groups were examined as separate groups given that they are considered different diagnostic entities based on DSM-5. Two-tailed independent samples *t*-test was used to assess any difference in average sleep quality between the clinical and control groups. Because of the relatively small sample size of each of the clinical subgroups and the exploratory nature of the study, significance was defined as *p*<.05. These statistical analyses were done using SPSS version 24 (IBM).

We used the statistical technique of partial least squares (PLS) [24,25] to examine whether the following variables were statistically predictive of sleep quality: age, gender, duration of BFRB illness, MGH-HPS total score, SPS-R total score, PSS total score, DTS total score, ADHD symptoms, BIS-11 total score, and SSRT. PLS is a particularly useful and robust statistical approach when variables are expected to be inter-correlated with each other [24,25]. Analysis was conducted using JMP Pro software version 13.0. Per convention, only explanatory variables with variable importance parameter >0.8 were retained. Variable importance parameter is a metric used to select a subset of relevant statistical predictors correlated with the response variable. It enables model estimation accuracy to be maximized, and models to be simplified, by excluding unimportant variables that do not contribute meaningfully to the predictive model.

## 3 Results

### 3.1 Participant characteristics

The sample consisted of 69 clinical participants (mean = 31.4 ± 9.0; 91.3% female) and 18 non-affected controls (mean age = 26.1 ± 5.34; 77.8% female). The large percentage of females is expected in study participants with these disorders. Participants had the following self-identified racial breakdown with no significant differences in demographic indicators between groups: 75.9% White/Caucasian, 4.6% Black/African-American, 5.7% Asian, 5.2% Biracial, and 3.7% Other. The group did not differ significantly on the above demographic measures (all *p*>.10).

Of the clinical subjects, 37 had a primary TTM diagnosis (mean age = 31.3 ± 8.2; 91.9% female). These participants reported a mean age of TTM onset of 11.7 (4.7) years. TTM participants had a mean score of 16.5 (4.4) [range 9 to 26] on the MGH-HPS. The remaining 32 had a primary diagnosis of SPD (mean age = 31.5 ± 9.9; 90.6% female). These participants reported a mean age of SPD onset of 13.5 (8.9) years. SPD participants had a mean score of 15.3 (5.0) [range 8 to 24] on the SPS-R.

### 3.2 Sleep quality

A statistically significant difference was found in PSQI global score between clinical participants (mean = 6.16; SD 3.04) and controls (mean = 4.17; SD 3.03) (t(84)=2.480, *p*=.015; d = 0.66). While the PSQI global score was numerically higher in individuals with TTM (mean = 6.49; SD = 2.97) than in SPD (mean = 5.77; SD = 3.12), this difference was not significant; t(66)=0.963, *p*=.339. Both clinical subgroups had, on average, a PSQI global score that indicated “poor sleep quality” according to the PSQI cutoff of 5 or higher.

Other clinical measures are presented in Table 1. Variables marked with an asterisk (*) were significantly different (*p*<.01) when compared between the clinical sample and the non-affected controls.

**Table 1:** Results of clinical assessments.

Assessment	Clinical Sample	TTM	SPD	Non-affected controls
MGH-HPS	–	16.51 ± 4.43	–	–
SPS-R	–	–	15.52 ± 5.14	–
PSS*	20.21 ± 7.04	20.11 ± 6.76	20.33 ± 7.48	11.53 ± 5.45
BIS11	63.15 ± 10.06	63.45 ± 11.01	62.79 ± 9.03	57.94 ± 64.99
DTS*	3.38 ± 0.93	3.37 ± 0.95	3.39 ± 0.92	4.16 ± 0.53
SSRT	221.82 ± 105.08	236.50 ± 119.08	203.09 ± 82.19	206.36 ± 64.99
Current comorbid conditions*	41 (59.42%)	19 (51.35%)	22 (68.75%)	1 (5.56%)
Major depressive disorder*	28 (40.58%)	12 (32.43%)	16 (5.00%)	1 (5.56%)
Any anxiety disorder	18 (26.09%)	8 (21.62%)	10 (31.25%)	0 (0.00%)
Any substance use disorder	6 (8.70%)	4 (10.81%)	2 (6.25%)	0 (0.00%)

MGH-HP = Massachusetts General Hospital Hairpulling Scale; SPS-R= Skin Picking Scale-Revised; PSS = Perceived Stress Scale; BIS11= Barratt Impulsiveness Scale 11; DTS = Distress Tolerance Scale; SSRT=Stop Signal Reaction Time.

Partial least squares (PLS) yielded an optimal 1-factor solution accounting for 36.5% of variance in the explanatory variables, and 43.2% of variance in sleep quality (Table 2). The PLS model indicated that the following variables were associated with worse sleep quality in patients: older age, worse hair pulling severity (MGH-HPS), higher levels of perceived stress (PSS), lower distress tolerance (DTS), higher impulsivity both on the BIS-11 and SST, and number of comorbid mental disorders. The following variables were not significantly related to sleep quality in the PLS model: gender, duration of illness, and SPS-R total scores (i.e. variable importance parameters were <0.8).

**Table 2:** Results of the PLS model. Positive coefficients indicate positive relationship with worse sleep.

Coefficient	
Current age, years	0.1681
MGH-HPS total score	0.1425
PSS total score	0.1859
DTS total score	−0.1503
BIS11 total score	0.1287
SSRT (msec)	0.1334
Number of comorbid mental disorders	0.1817

MGH-HPS = Massachusetts General Hospital Hairpulling Scale; PSS = Perceived Stress Scale; BIS11= Barratt Impulsiveness Scale 11; DTS = Distress Tolerance Scale; SSRT=Stop Signal Reaction Time.

## 4 Discussion

In this study, we examined BFRBs and their relationship to sleep quality in a sample of adults. We hypothesized that BFRBs would be associated with poorer sleep quality than controls and that poorer sleep quality would be associated with greater BFRB symptom severity. Supporting our hypothesis, we found that those with a BFRB had significantly worse sleep quality than controls (with a medium effect size). Furthermore, our hypothesis that sleep quality would be associated with worse symptom severity was partially supported. Severity of TTM was associated with poor sleep quality but not skin picking severity. These findings are somewhat consistent with prior findings [26] in which subjects with BFRBs reported more sleep difficulties than non-affected controls, but they also highlight the fact that poor sleep in TTM and SPD may be driven by somewhat different mechanisms.

We previously reported that sleepiness problems among non-clinical young adults were associated with higher scores on the self-report BIS-11 [27]. The current study furthers that association by finding that poor sleep was also significantly associated with the SSRT. Previous research suggests that the BIS and SSRT are somewhat dissociable and in fact may reflect distinct latent factors of impulsive action and impulsive personality traits, respectively [28]. Based on this previous research, the current findings demonstrate an association between sleep problems in BFRBs and both traits of impulsivity as well as impulsive action.

To put the BFRB sleep quality results into perspective, these data show that subjective sleep quality was poor, but not dramatically so (with a mean score of approximately 6 – higher scores indicate worse sleep quality). Data using this same scale in other psychiatric disorders have shown that subjective sleep quality is notably worse in major depressive disorder (mean scores of 10–12), PTSD (mean scores 10–11), chronic pain patients (mean score of 8), and obsessive compulsive disorder (mean score of 8) [[29], [30], [31]]. Interestingly, and perhaps relevant to BFRBs, the limited research in the field of OCD suggests that sleep disturbances may contribute to the illness [32]. A recent study found that individuals with OCD plus a sleep disturbance reported increased OCD symptoms compared to individuals with OCD and no sleep disturbance, thereby suggesting even minor sleep disturbance may increase the severity of OCD symptoms [33]. So, even if the subjective sleep quality is not as high as seen in other disorders, the poor sleep associated with BFRB may still impact the overall symptom severity.

What this study also adds to the literature is the finding that perceived stress, distress intolerance, and impulsivity (i.e. reflected by both the SSRT and the BIS) all statistically contribute, at least to some degree, to impaired sleep in BFRBs. For all of these variables, causality is still uncertain and the effects fairly small. First, it is possible that those with BFRBs experience worse sleep due to being up at night pulling or picking, distressed by other issues, and being unable to calm down one's impulsive nature. Alternately, and not mutually exclusively, poor sleep could lead to worse symptoms, more distress and perceived stress, and more impulsivity by mechanisms not yet fully understood. These associations appear further complicated by the fact that directionality may differ based on which variable is examined. Thus, increased distress could result in poor sleep which in turn could lead to an inability to control impulsive behavior such as pulling and picking. Interestingly, there are data to suggest that last night's sleep quality predicts next day anxiety, OCD symptoms, and PTSD, but not vice versa [[34], [35], [36]]. Whether these findings are the same for BFRBs awaits more detailed examination.

What possible physiological mechanisms may be involved in the relationship between poor sleep quality and BFRBs remains unknown. There are several possible, non-mutually exclusive, theories. First, research suggests that poor quality sleep may result in a hyperactive limbic response to negative motional stimuli with a loss of functional connectivity with the medial prefrontal cortex and thus a problem with top-down, prefrontal control [37]. This could possibly explain the association between impulsivity, increased stress/poor distress tolerance and BFRB symptom severity seen in our study. Second, poor quality sleep has been associated with alterations in the hypothalamic–pituitary–adrenal (HPA) pathway and cortisol levels (i.e. a type of stress response) (for a review see [38]). This in turn may also affect subsequent stress reactivity and therefore explain the behaviors of pulling and picking as ways of coping with stress. Third, recent research identified common structural brain abnormalities associated with both ADHD and sleep problems – specifically, common structural changes in the ventral attentional system and fronto-striatal circuitry [39]. Thus, in the context of sleep problems and BFRBs, the possibility also exists that common abnormalities in such brain circuits could explain the association with sleep problems. This would be of interest to examine in future imaging work in BRFBs. Despite not knowing directionality in our study, the clinical importance of these associations is that sleep may play a large and yet understudied role in helping people with their BFRBs. Instead of, or in addition to, focusing on other psychological variables, sleep should be assessed and perhaps receive focus as a potential treatment target, instead of simply being seen as a consequence of the other variables. This may be even more important in younger people with BFRBs as longitudinal research in other mental areas has shown that poor sleep may be predictive of later age anxiety problems [40]. More research will need to be done to ascertain the answer to these questions, using longitudinal designs and/or mediation analyses in BFRB cohorts.

The findings from this study show an association between sleep problems and TTM symptom severity; as well as between sleep problems and particular traits (perceived stress, distress intolerance, and impulsivity), in people with BFRBs. Therefore, our research suggests that these factors may be important for clinicians to address when treating patients with SPD or TTM. In addition to other commonly addressed variables, it may also be valuable for clinicians to assess sleep quality and focus on improving sleep quality in patients as a treatment add-on to other behavioral interventions. Additionally, our results suggest that using techniques to enhance coping with stress or decreasing impulsivity in BFRB patients could offer possible benefit as well. A potentially viable treatment may be crafted using certain facets of dialectical behavior therapy (DBT). Previous studies utilizing this method in TTM treatment showed significant positive long-term effects [41,42]. Therefore, it may be beneficial to tailor treatment to emulate some of the key aspects of DBT, including mindfulness, distress tolerance, and emotion regulation. This may be especially helpful in combination with improving sleep hygiene.

While this study sheds light on sleep dysfunction in BFRBs and implicated traits, there are limitations to the study. First, the sample size was relatively small and included mostly females. Second, this study was conducted solely using data from adult participants. Therefore, it may be beneficial to capture and analyze data regarding sleep and symptom severity in children and adolescents with BFRBs. Though as expected comorbid mental disorders in patients was associated with worse sleep, above and beyond the impact of other variables, the sample size was not adequate to address the impact of particular comorbidities on sleep. Furthermore, the PSS and BIS-11 demonstrated fairly low reliability in this sameple. In addition, another potential limitation is the lack of experimental manipulation of sleep and the possibility that shared method variance (i.e., self-report) could theoretically have inflated estimates; though this seems unlikely given that only some variables were associated with poor sleep. Future research may be wise to perform actual in-laboratory sleep assessments to more objectively measure sleep problems in BFRBs. Addressing these would allow clinicians to have a more comprehensive understanding of the relationship between sleep and BFRBs.

## 5 Conclusions

The current study is the first to assess sleep quality and its associations with symptom severity, perceived stress and impulsivity in adults with TTM and SPD. The data suggest that those with BFRBs have significantly poorer sleep quality than unaffected healthy adults. Additionally, we found in people with BFRBs that hair pulling symptom severity has a significantly negative relationship with sleep quality, but not skin picking severity; and that poorer sleep quality was related to impulsivity, perceived stress, distress intolerance, and comorbid mental disorders, across patients with BFRBs examined. In light of these findings, treatment should take this relationship into account and further research should be done to address the limitations of this study, as well as to ascertain whether this relationship is causational or purely correlational.

## Funding

This study was funded by the TLC Foundation for Body-Focused Repetitive Behaviors.

## Disclosures

Dr. Grant has received research grants from the TLC Foundation for Body-Focused Repetitive Behaviors, the American Foundation for Suicide Prevention, Takeda, Neurocrine, Biohaven, and Avanir Pharmaceuticals. He receives yearly compensation for acting as editor-in-chief of the Journal of Gambling Studies and has received royalties from Oxford University Press, American Psychiatric Publishing, Inc., Norton Press, and McGraw Hill. Dr. Grant has received a research grant from the TLC Foundation for Body-Focused Repetitive Behaviors. Ms. Cavic and Ms. Valle report no financial relationships with commercial interests. Dr. Chamberlain consults for Ieso and Promentis; and receives a stipend from Elsevier for editorial work. Dr. Chamberlain's role in this study was funded by a Wellcome Trust Clinical Fellowship (110049/Z/15/Z).
